# Silicon Nitride as a Biomedical Material: An Overview

**DOI:** 10.3390/ijms23126551

**Published:** 2022-06-11

**Authors:** Xiaoyu Du, Seunghun S. Lee, Gurdial Blugan, Stephen J. Ferguson

**Affiliations:** 1 Institute for Biomechanics, ETH Zurich, 8093 Zurich, Switzerland; seunglee@ethz.ch (S.S.L.); sferguson@ethz.ch (S.J.F.); 2 Laboratory for High Performance Ceramics, Empa, Swiss Federal Laboratories for Materials Science and Technology, 8600 Dübendorf, Switzerland; gurdial.blugan@empa.ch

**Keywords:** silicon nitride, mechanical properties, osteogenesis, antibacterial, implants

## Abstract

Silicon nitride possesses a variety of excellent properties that can be specifically designed and manufactured for different medical applications. On the one hand, silicon nitride is known to have good mechanical properties, such as high strength and fracture toughness. On the other hand, the uniqueness of the osteogenic/antibacterial dualism of silicon nitride makes it a favorable bioceramic for implants. The surface of silicon nitride can simultaneously inhibit the proliferation of bacteria while supporting the physiological activities of eukaryotic cells and promoting the healing of bone tissue. There are hardly any biomaterials that possess all these properties concurrently. Although silicon nitride has been intensively studied as a biomedical material for years, there is a paucity of comprehensive data on its properties and medical applications. To provide a comprehensive understanding of this potential cornerstone material of the medical field, this review presents scientific and technical data on silicon nitride, including its mechanical properties, osteogenic behavior, and antibacterial capabilities. In addition, this paper highlights the current and potential medical use of silicon nitride and explains the bottlenecks that need to be addressed, as well as possible solutions.

## 1. Introduction

Silicon nitride (Si_3_N_4_) was first synthesized in 1859 and is a relatively new artificial material [1]. As a synthetic non-oxide ceramic, Si_3_N_4_ is considered a ceramic-glass composite because it contains crystalline grains and amorphous grain-boundary phases [2]. Due to its excellent mechanical properties, it was initially used in industrial applications such as ball bearings, cutting tools, sealing elements, and thermal machine components [3,4,5]. Additionally, Si_3_N_4_ has desirable thermomechanical and tribological properties and provides good performance in terms of hardness and fracture toughness, which are strictly required for high-load medical applications [6]. Due to its good mechanical properties and biocompatibility, Si_3_N_4_ has been considered as a potential biomedical material since the 1980s, especially for orthopedic applications. To date, several Si_3_N_4_ spinal spacer and intervertebral fusion cage products have been cleared by the CE and FDA, based on animal studies and standard compliance requirements. Meanwhile, it has been extensively studied as a bearing that can improve the longevity of knee joints and prosthetic hips [7]. Moreover, Si_3_N_4_ is non-ferrous, non-electromagnetic, and partially radiolucent that minimizes scatter and associated artifacts on computerized tomography (CT) and magnetic resonance imaging (MRI) in patients with Si_3_N_4_ implants [8].

Si_3_N_4_ can be considered as a class of materials comparable to steel due to their excellent mechanical properties. In particular, in terms of compressive strength, Si_3_N_4_ exhibits relatively high values (~4000 MPa) that exceed even the metallic materials used for implants (i.e., 600–1800 MPa for cobalt chromium and 800–970 MPa for Ti6Al4V) [9]. While most ceramics are brittle, Si_3_N_4_ can resist brittle fracture due to its relatively high toughness. In addition to high fracture toughness, Si_3_N_4_ has high wear resistance and a low coefficient of friction [10]. The size and shape of Si_3_N_4_ grains, as well as the amount and chemical composition of the grain boundary phases, are key parameters that guide the mechanical behavior of Si_3_N_4_. For instance, Si_3_N_4_ with high fracture toughness usually has coarse grains, while high-strength Si_3_N_4_ possesses an elongated microstructure with fine grains [11]. The mechanical properties of Si_3_N_4_, such as fracture toughness, hardness, and compressive strength, have been systematically investigated over the past decades. 

Biomedical materials should not only have excellent mechanical properties but also a favorable response to the physiological environment. Initially, there was controversy about the biocompatibility of Si_3_N_4_. Some early researchers assumed that non-oxide ceramics could not achieve long-term biocompatibility due to the additives used in the sintering process [12]. However, in the last decades, a large number of reports demonstrated the biocompatibility and even the bioactivity of Si_3_N_4_ [13,14,15]. Nowadays, it is widely accepted that Si_3_N_4_ can accelerate bone repair and induce osseointegration. Another favorable property of Si_3_N_4_ for medical applications is its antibacterial ability, which is essential for orthopedic implants.

Although Si_3_N_4_ has been intensively studied for years as a biomedical material, there is a paucity of comprehensive data on its properties and medical applications. In this review, we will elucidate the mechanical, osteogenic, and antibacterial properties of Si_3_N_4_, which will be of great interest to professionals in the medical and engineering fields. Finally, we will present the current clinical and research landscape of Si_3_N_4_ implants. By understanding the properties of Si_3_N_4_, we can find additional ways to enhance its potential for clinical applications.

## 2. Synthesis and Manufacturing Process of Si_3_N_4_

There are several methods used to produce Si_3_N_4_, including reaction-bonding [16], hot-pressing [17,18,19], hot-isostatic pressing [20,21,22], pressureless sintering [23,24,25], and gas-pressure sintering techniques [26,27,28]. Reaction-bonded Si_3_N_4_ (RBSN) is made by nitriding a porous silicon (Si) compact in the temperature range of 1200—1500 °C [16]. A consequence of this reaction is that Si_3_N_4_ grows in the porosity of the compact; therefore, the reaction-bonded product has nearly the same external geometry and dimensions as the molded Si article, without subsequent grinding and additional manufacturing costs. Nevertheless, the resulting bodies contain relatively high porosity (typically 15–20%) [7], which reduces specific mechanical properties, such as fracture toughness and flexural strength. Hot-pressing and hot-isostatic-pressing are two popular pressure-assisted methods, dating back to the 1960s. Hot-pressed Si_3_N_4_ is achieved by adding a flux such as magnesia to a fine Si_3_N_4_ powder and then pressing the powder into a graphite die at high temperatures (typically >1700 °C) and pressures (40—50 MPa). Likewise, hot-isostatic pressing (HIP) is similar to hot-pressing, but it requires the encapsulation of the powders and higher pressure (typically 150—200 MPa). The final products produced by both techniques are fully dense with favorable mechanical properties. Nevertheless, the fabrication cost is relatively high, and it is only suitable for simple shapes. Subsequently, a cost-effective method is to sinter Si_3_N_4_ to a stage where the pores become isolated and then hot-isostatically press it to obtain the high-strength ceramic [7]. In addition, Si_3_N_4_ can also be densified by pressureless sintering in a nitrogen environment at around 1750 °C. 

Si_3_N_4_ is essentially a covalent compound with very low bulk diffusion, which makes it difficult to consolidate [11]. Hence, it is usually sintered with small amounts of oxide sintering additives such as yttrium oxide (Y_2_O_3_) and aluminum oxides (Al_2_O_3_), which invoke a liquid-phase sintering mechanism by forming a eutectic liquid with the oxidized surface layer of the Si_3_N_4_ powder, thereby densifying the ceramic and enhancing its oxidation resistance [29]. Nevertheless, the liquid phase produced by the sintering additive is usually retained in glassy intergranular phases, leading to the deterioration of mechanical properties [30]. Therefore, a lot of effort has been made to reduce the detrimental effects of the glassy phase, such as transient liquid-phase sintering, increasing the refractoriness of the boundary phase, and post-sintering heat treatments [30]. 

**Figure 1:**
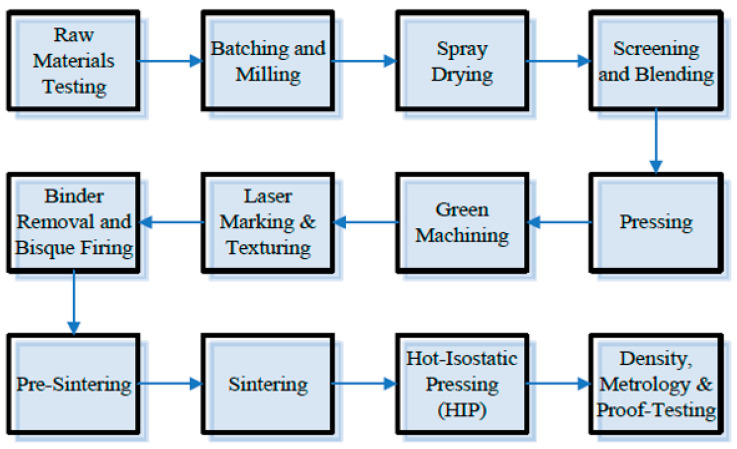
One example of a manufacturing process flow diagram for the production of biomedical Si_3_N_4_ intervertebral spinal spacers. The image is printed with permission from ref. [31].

To further understand the manufacturing process of Si_3_N_4_ biomedical implants, Figure 1 is a flow diagram of a manufacturing process for the production of Si_3_N_4_ intervertebral spinal spacers at SINTX Technologies Corporation (Salt Lake City, UT, USA). Briefly, green components consisting of intricately mixed Si_3_N_4_, Y_2_O_3_, and Al_2_O_3_ raw materials are placed in an ambient N_2_ continuous furnace at a temperature > 1700 °C. This pressureless process step densifies the parts to a state of closed porosity. Then, hot-isostatic pressing (HIP) is performed to obtain further densification. Commonly used parameters are set at >1650 °C and >200 MPa of nitrogen. The resulting Si_3_N_4_ samples consist of a two-phase microstructure with anisotropic β-Si_3_N_4_ grains separated by continuous SiYAlON grain boundaries [31]. 

Typically, as-fired Si_3_N_4_ has a large number of acicular grains protruding from its surface. This provides the ceramic with high wettability, but its crystallographic structures and the amount of dissoluble surface ions may vary. Moreover, it can be manufactured into dense or porous products according to different demands. In addition, the properties of Si_3_N_4_ can vary considerably depending on different forming routes. By controlling the composition of the starting material, the type of sintering aids, and the heat treatment parameters, Si_3_N_4_ with specific properties can be obtained. Importantly, the surface of Si_3_N_4_ can be modified by either polishing or roughening. Its surface chemistry can also be extensively modulated from a surface consisting mainly of silicon-amines (Si-NH_2_) to a silica (SiO_2_) [32,33]. Note that both surface morphology and stoichiometry have a crucial influence on the relevant mechanical and biological properties of Si_3_N_4_. For instance, it was reported that the properties of Si_3_N_4_ at room temperature are controlled by the size and aspect ratio of the β-phase grains, while its strength at high-temperatures is mainly dependent on the grain boundary phase [11,34,35].

## 3. Mechanical Properties of Si_3_N_4_

### 3.1. Basic Mechanical Properties

The basic mechanical properties of ceramics are fracture strength, fracture toughness, and fatigue resistance. The dynamic failure process of ceramics can spread the impact load over a larger area and extend the time of impact loading, thus reducing the stress on the backing structure [36]. Material properties, confinement, geometry, and interface conditions are important parameters that influence the failure of a ceramic. Si_3_N_4_ has excellent mechanical properties. Its flexural strength is between 800 and 1100 MPa, and the elastic modulus is in the range 296–313 GPa [9]. The fracture strength of Si_3_N_4_ can be modulated by controlling the size and amount of large elongated grains (β-Si_3_N_4_) in a fine-grained matrix. Like whisker-reinforced ceramics, β-grains form elongated hexagonal prisms that bridge advancing cracks, placing the crack-tips under compression [37]. The ratio of α-Si_3_N_4_ to β-Si_3_N_4_ in the starting powder; the additives used for densification; the sintering temperature; and the holding time have important effects on the extent of elongated grain growth. Becher et al. [38] prepared Si_3_N_4_ samples with different microstructures by gas pressure sintering and hot pressing with the addition of elongated β-Si_3_N_4_ seeds. The average size of the grains increased with temperature. However, the additive composition did not show a significant effect on the glass viscosity and diffusion rate. The results illustrated that microstructures with wide grain diameter distribution or fine equiaxed microstructures were not beneficial for improving fracture strength. In contrast, the presence of large, elongated grains, especially microstructures with a distinct bimodal grain distribution, contributed to improved fracture resistance. The specific experimental data are shown in Table 1. 

Besides fracture strength, fracture toughness is another main metric for evaluating the ability of Si_3_N_4_ to resist crack propagation. The fracture toughness of Si_3_N_4_ usually ranges between 5.0 MPa/m^2^ and 7.0 MPa/m^2^. However, this value will vary due to different processing methods and disparate test techniques [39,40]. It is well known that the fracture toughness of Si_3_N_4_ relies on its grain morphology. The large elongated β-phase grains within a fine-grained matrix contribute to improved fracture toughness [41]. Chen et al. [42] used a controlled-flaw in combination with the miniaturized disk-bend test (MDBT) methods to evaluate the fracture toughness of Si_3_N_4_. It is worth noting that MDBT is also suitable for testing diminutive specimens (i.e., disks around 3 mm in diameter and 250 μm thick). The crack resistance value as determined by MDBT was 6.53 ± 0.88 MPa/m^2^, which coincides with conventional measurements. 

Like all ceramics, Si_3_N_4_ is inherently brittle and sensitive to flaws. Microcracks generated under thermal shock and dynamic loads need to be controlled in order to improve its reliability. Uniquely, Si_3_N_4_ has the capability of crack-healing during its high-temperature use [43]. Several researchers found that Si_3_N_4_ can exhibit crack-healing at high temperature due to the fact that the oxidation products react with the matrix and produce secondary phases that provide strong bonding between the crack walls [44,45]. For example, Choi and coworkers [46] compared the strength of pre-cracked samples after annealing at high temperatures in air and nitrogen atmospheres. They found that the strength of Si_3_N_4_ increased significantly at temperatures between 800 °C and 1200 °C in air as a result of crack-healing. However, in nitrogen, although the strength of the annealed samples at above 1200 °C increased, this may be due to the release of residual contact stresses rather than crack healing. Another work indicated that annealing at 1200 °C for 100 h increased the strength of Si_3_N_4_ samples compared to identical samples annealed for 30 min [47]. However, this does not mean that higher temperatures and longer times always contribute to crack-healing. Easler et al. [48] found that the strength of Si_3_N_4_ decreased for longer times up to 50 h in comparison with those for 30 min at the same temperature of 1370 °C.

Due to its excellent mechanical properties, Si_3_N_4_ has also been used as a strengthening agent for other bioactive ceramics [49]. For instance, Amaral et al. [6] pursued suitable conditions to fabricate almost fully dense silicon-nitride biocomposites by hot-pressing with optimized parameters (1350 °C—40 min—30 MPa). The presence of Si_3_N_4_ enhanced the mechanical properties of the bioglass. Remarkably, the new biomaterial composites showed a significant improvement in bending strength (383 ± 47 MPa) and fracture toughness (4.4 MPa/m^2^) compared to the pure bioglass. Another study reported that graded glass/ceramic materials exhibited a unidirectional gradient in elastic modulus from the surface to the interior when the contact surface of the dense, high modulus Si_3_N_4_ was infiltrated into the lower modulus silicon oxynitride glass. In addition, the surface of this graded Si_3_N_4_ provided significantly better resistance to contact damage than either constituent glass or ceramic [50].

In one of our studies [51], we evaluated the correlation between mechanical properties and the porosity of Si_3_N_4_ bioceramics. Our results showed that Young’s modulus and compressive strength decreased non-linearly with increasing porosity. Furthermore, the Young’s modulus of a Si_3_N_4_ beam with 70% porosity (26.26 ± 1.23 GPa) was 11 times lower than that of a dense Si_3_N_4_ beam (298.45 ± 1.08 GPa), while the fracture toughness only decreased 5 times. The fracture toughness (1.06 ± 0.06 MPa/m^2^) and the compressive strength (100.35 ± 3.39 MPa) of the porous Si_3_N_4_ were sufficient to serve as a load-bearing substitute for trabecular bone.

Notwithstanding the foregoing, the research on the impact and frequency-dependent resonance of Si_3_N_4_ is far from being extensively developed, especially under dynamic loading conditions. This would be a meaningful and original topic to comprehensively study.

### 3.2. Methods to Enhance the Mechanical Properties of Si_3_N_4_

Unlike polymers whose mechanical properties can be varied by tuning monomer length and crosslink density, bulk Si_3_N_4_ has a well-defined stiffness that can only be varied within a relatively small range. However, its hardness and fracture toughness can be affected by microstructural characteristics, including composition, porosity, the content of intergranular phases, and the shape and size of its grains. For instance, in-situ reinforced Si_3_N_4_ that has β-Si_3_N_4_ grains with high aspect ratios consistently shows better mechanical properties in terms of fracture, creep resistance, and strength. Many methods have been applied to further enhance its mechanical properties, including tailoring sintering parameters, adding specific sintering additives, ion implantation, and adding reinforcement agents. 

The formation of sub-surface voids is detrimental to the mechanical properties, which are largely determined by processing methods [52]. The different sintering techniques steer the mechanical behavior of Si_3_N_4_. Benjamin and colleagues [53] compared the mechanical properties of Si_3_N_4_ consolidated by two sintering methods, hot-pressing and pressureless sintering. The sintered samples were designated as HPSi_3_N_4_ and LPSSi_3_N_4_, respectively. HPSi_3_N_4_ showed higher density, hardness, and characteristic strength but lower fracture toughness in comparison with LPSSi_3_N_4_. The detailed data are shown in Table 1. Moreover, the strengths of both HPSi_3_N_4_ and LPSSi_3_N_4_ are significantly higher than the common oxide ceramics alumina and zirconia. This example indicated that sintering parameters and techniques also affect the mechanical properties of Si_3_N_4_ and that users can choose suitable methods to obtain products with desired performance. 

The sintering additive system of Al_2_O_3_-Y_2_O_3_ is effective in inducing excellent mechanical strength in Si_3_N_4_. However, it requires high nitrogen pressure and sintering temperature. On the other hand, alkaline earth oxides (i.e., SrO-MgO-CeO2) as new sintering additives were utilized to densify Si_3_N_4_ at a lower sintering temperature and atmospheric nitrogen pressure [54]. Pressureless sintering of Si_3_N_4_ doped with a combination of MgO and Y_2_O_3_ has been successfully achieved by Ling et al. [55]. In their study, 5 wt.% MgO + (0, 1, 2, 3, 4, 5, and 6 wt.% Y_2_O_3_) was doped into Si_3_N_4_. It was found that the combination of MgO and Y_2_O_3_ was very effective since both react with the SiO_2_ on Si_3_N_4_ particles to form a silicate liquid phase that promotes densification during sintering. The amount of Y_2_O_3_ affected the mechanical properties of the sintered ceramic. The composition of Si_3_N_4_ + 5 wt.% MgO + 4 wt.% Y_2_O_3_ (sintered at 1700 °C for 60 min) showed the most favorable properties with a high density up to 99%, a bending strength of 950 MPa, and a fracture toughness of 7.5 MPa/m^2^. Liu et al. [56] prepared pressureless sintered Si_3_N_4_ using a sintering additive from the MgO - Al_2_O_3_ - SiO_2_ system by conventional and high-energy planetary ball-milling. For Si_3_N_4_ fabricated by planetary ball-milling, they showed an improvement in the density and homogeneity of the sintering additives, which may lead to an improvement in the mechanical properties. Using this method, pressureless sintered Si_3_N_4_ with a hardness of 14.2 GPa, a flexure strength of 1.06 GPa, and a fracture toughness of 6.4 MPa/m^2^ was achieved. This was higher than the values achieved by traditional ball-milling and comparable to Si_3_N_4_ prepared by hot-pressing or gas pressure sintering. 

**Figure 2:**
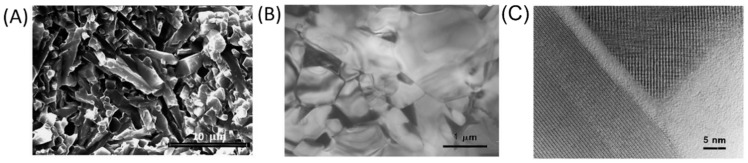
(**A**) An SEM image of the specimen prepared in Ref [30]. The Si_3_N_4_ grains show high aspect ratios and form an interlocking structure. (**B**) Low-magnification TEM micrographs of the materials. (**C**) High-resolution TEM images of the amorphous phase at the grain pockets. This image is reprinted with permission from Ref. [30]. Copyright 1998 Elsevier Publishing Group.

Rare-earth oxide additives have also been added to Si_3_N_4_ to obtain specific microstructures and improve mechanical properties [57]. For instance, Liu et al. [30] prepared in-situ reinforced Si_3_N_4_ via gas pressure sintering with La_2_O_3_, Y_2_O_3_, and SrO additives. Two crystalline rare-earth apatite phases, Y_5_Si_3_O_12_N and La_5_Si_3_O_12_N, were detected at grain boundaries with a thickness of approximately 1.7 nm. Amorphous phases also appeared at the two-grain boundaries, with thicknesses ranging from 0.7 to 3.0 nm due to incomplete recrystallization. This was found to be beneficial for superior high-temperature strength. In this sintering system, a complete transformation from α-Si_3_N_4_ to β-Si_3_N_4_ was successfully achieved, and the average grain diameter was circa 0.8 µm, with an aspect ratio greater than 4. Moreover, as shown in Figure 2A, there was an interlocking microstructure that was resistant to crack propagation. Under compressive loads, the acicular grains interlock and can inhibit grain-boundary sliding, thus improving creep resistance. In addition, the elongated grains can contribute to the stress rupture performance via the bridging of microcracks.

Ytterbium oxide (Yb_2_O_3_) is another effective sintering additive to improve the mechanical properties of Si_3_N_4_. Lee et al. [58] fabricated gas-pressure-sintered Si_3_N_4_ with 4 wt.% Yb_2_O_3_ as a sintering aid and performed microstructural evaluation and mechanical tests. However, the microstructure of the entire specimen was heterogeneous. Small grains were formed in the inner regions of specimens, while extremely large, elongated grains appeared in a fine matrix in the outer regions near the surface. Interestingly, the strength of the inner region was higher than that of the outer region. However, the fracture toughness and R-curve behavior of the inner region were not as good as those of the outer region. These variations in microstructure have a strong influence on mechanical properties. Therefore, researchers can utilize this knowledge to fabricate functionally graded microstructures by controlling the relative thickness of each region. Later, Silva et al. [59] prepared two different compositions of Si_3_N_4_ with microstructures consisting of grains of β-Si_3_N_4_ distributed in the secondary phase. The SN1 samples (with a composition of 91 wt.% Si_3_N_4_, 3 wt.% Al_2_O_3_, 3 wt.% Y_2_O_3_, and 3 wt.% Yb_2_O_3_) achieved a hardness of 11.1 ± 0.2 GPa and a fracture toughness of 5.0 ± 0.4 MPa/m^2^. In contrast, SN4 samples (with a composition of 90 wt.% Si_3_N_4_, 6 wt.% Al_2_O_3_, and 4 wt.% Y_2_O_3_) achieved a hardness of 13.2 ± 0.2 GPa and fracture toughness of 4.5 ± 0.2 MPa/m^2^. Although the fracture toughness of both compositions was similar, the SN4 achieved higher hardness. 

In addition, SiC nanoparticles have been used as additives to improve the mechanical properties of Si_3_N_4_. Li et al. [35] utilized microwave sintering, which promotes the rapid densification and refinement of the crystalline microstructure. The addition of the SiC nanoparticles not only strengthened the ceramic but also promoted the α→β phase transformation of Si_3_N_4_. Therefore, in their study, the Si_3_N_4_/n-SiC ceramics (85 wt.% Si_3_N_4_ + 5 wt.% n-SiC + 5 wt.% Al_2_O_3_ + 5 wt.% Y_2_O_3_) at a sintering temperature of 1600 °C and a holding time of 10 min exhibited the best mechanical properties, with the density being 97.1%, the hardness being 14.44 GPa, and a fracture toughness of up to 7.77 MPa/m^2^. These results were 2.8%, 7.0%, and 13.1% higher than the Si_3_N_4_ without SiC nanopowders, respectively.

In addition to various sintering additives, Shi et al. [60] used ion implantation to improve the bending strength of Si_3_N_4_. However, their results showed that the strength initially augmented with small doses of implanted metal ions (i.e., Ti, Zr, and Cr) decreased with higher implantation doses. The increase in bending strength was attributed to the generation of tough phases such as Zr_2_Si or Ti_2_Si. However, high implanted doses made the ceramic amorphous, thus leading to the relaxation of residual stress. Si_3_N_4_ composites reinforced with two kinds of graphene fillers (nanoplatelets and reduced graphene oxide sheets) and consolidated by spark plasma sintering were prepared by Seiner et al. [61]. The elastic constants (c_ij_) of these blends were examined by resonant ultrasound spectroscopy. Then, the Young’s modulus and the shear modulus in different directions were calculated based on the coefficients c_ij_. The results illustrated that Si_3_N_4_ composites showed enhanced anisotropic behavior regarding elastic constants when the fillers were fully contacted. With the increasing filler contents, the elastic and shear moduli steadily decreased. This significant softening of E and G proved that the toughening effect of the fillers could restrain tension-induced fracture in given directions. Mauro et al. [62] designed and produced three different Si_3_N_4_-based ceramics: (A) Si_3_N_4_ + 10 vol.% bioactive glass; (B) Si_3_N_4_ + 8.6 vol.% MgO; and (C) Si_3_N_4_ + (2 wt.% Y_2_O_3_ + 5 wt.% Al_2_O_3_) + 35 vol.% TiN. As for the hardness and Young’s modulus of the material, sample A composed of the addition of 10 vol.% bioactive glass was smaller due to the presence of an amorphous phase. In contrast, the modulus of the Si_3_N_4_-TiN composite was the highest compared to the other two types since the addition of TiN reinforced the stiffness of the matrix. More importantly, Si_3_N_4_-TiN was also confirmed to be electroconductive. These properties allow for the fabrication of complex-shaped components (including clinical applications) by electrical discharge machining. In one of our studies [63,64], we investigated in more detail the fractography and mechanical properties of commercial Si_3_N_4_-titanium nitride composites. We found that the density, Young’s modulus, and fracture toughness of Si_3_N_4_-titanium nitride composite increased linearly with TiN content (0, 10, 20, and 30 wt.%). Moreover, an increase in strength was observed in composites with TiN content of 20 wt.% and 30 wt.%, and when 30 wt.% TiN was added, the fracture toughness increased by 11% compared to pure Si_3_N_4_ due to residual stresses in the Si_3_N_4_ matrix and titanium nitride particles.

**Table 1:** The mechanical properties of Si_3_N_4_ ceramics.

Author	Ref.	Materials	Remarks	Bending Strength (MPa)	Flexural Strength (MPa)	Vickers Hardness (GPa)	Young’s Modulus (GPa)	Weibull Modulus	Poisson’s Ratio	Fracture Toughness (MPa/m^2^)	Fracture Strength (MPa)
Chen et al.	[42]	Si_3_N_4_	Disks 3 mm in diameter and typically range in thickness from 250 to 400 μm	/	/	12–13	299	/	0.270	/	/
Silva et al.	[59]	Si_3_N_4_ (SN1)	Composition with 91 wt.% Si_3_N_4_, 3 wt.% Al_2_O_3_, 3 wt.% Y_2_O_3,_ and 3 wt.% Yb_2_O_3_	/	/	11.1 ± 0.2	Assuming 300	/	/	5.0 ± 0.4	/
		Si_3_N_4_ (SN4)	Composition with 90 wt.% Si_3_N_4_, 6 wt.% Al_2_O_3_, and 4 wt.% Y_2_O_3_	/	/	13.2 ± 0.2	Assuming 300	/	/	4.5 ± 0.2	/
Bal et al.	[64]	Si_3_N_4_	Composition with 90 wt.% Si_3_N_4_, 4 wt.% Al_2_O_3_, and 6 wt.% Y_2_O_3_.	/	923 ± 70	14–16	300–320	19	/	10 ± 1	/
Becher et al.	[38]	Si_3_N_4_	6.25 wt.% Y_2_O_3_ + 1 wt.% Al_2_O_3_. Average grain diameter: 0.3 mm/2 mm, distinctly bimodal	/	/	/	/	/	/	/	1144 ± 126
			6.25 wt.% Y_2_O_3_ + 1 wt.% Al_2_O_3_. Average grain diameter: ~0.4 mm, and monomodal								660 ± 165
			5 wt.% Y_2_O_3_ + 2 wt.% Al_2_O_3_. Average grain diameter: 10.3 mm/2 mm, distinctly bimodal								1011 ± 78
			5 wt.% Y_2_O_3_ + 2 wt.% Al_2_O_3_. Average grain diameter: 0.5 mm, monomodal with definite tail to larger sizes								660 ± 20
Liu et al.	[56]	Si_3_N_4_	Commercially available Si_3_N_4_ powder (E-10, UBE Industries, Japan) was mixed with 3 wt.% MgO (99.9%), 1.5 wt.% Al_2_O_3_ (99.9%), and 3.5 wt.% SiO_2_ (99.9%) additives.	/	1060	14.2	/	/	/	6.4	/
Ling et al.	[55]	Si_3_N_4_	Si_3_N_4_ + 5 wt.% MgO + 4 wt.% Y_2_O_3_ (sintered at 1700 °C during 60 min)	950	/	/	/	/	/	7.5	/
Li et al.	[35]	Si_3_N_4_/n-SiC	Si_3_N_4_ /n-SiC (85 wt.% Si_3_N_4_ + 5 wt.% n-SiC + 5 wt.% Al_2_O_3_ + 5 wt.% Y_2_O_3_) at sintering temperature of 1600 °C and holding time of 10 min	/	/	14.44	/	/	/	7.77	/
Benjamin et al.	[53]	Si_3_N_4_	HPSi_3_N_4_ (density 99.6%), hot pressing	/	/	1640 HV	/	10.7	/	6.2	1210
			LPSSi_3_N_4_ (density 96.7%), pressureless sintering			1565 HV		10.7		7.1	990
McEntire et al.	[31]	Si_3_N_4_	6 wt.% Y_2_O_3_, 4 wt.% Al_2_O_3_, 90 wt.% Si_3_N_4_	/	995	/	/	/	/	10.6	/
Mauro et al.	[62]	Si_3_N_4_ based composite	90 vol.% Si_3_N_4_ + 10 vol.% bioactive glass	/	934 ± 215.5	15.29 ± 0.51	276	/	/	/	/
			91.3 vol.% Si_3_N_4_ + 8.7 vol.% MgO		969 ± 149.2	18.70 ± 6.63	316				
			65 vol.% (93 wt.% Si_3_N_4_ + 2 wt.% Y_2_O_3_ + 5 wt.% Al_2_O_3_) + 35 vol.% TiN		835 ± 116.0	14.70 ± 0.70	354				
Blugan et al.	[63]	Si_3_N_4_ - Titanium Nitride Composites	Si_3_N_4_–TiN composites with 0 wt.% TiN	790 ± 122.0	/	/	303	/	/	4.26 ± 0.09	/
			Si_3_N_4_–TiN composites with 10 wt.% TiN	685 ± 51.6			311			4.47 ± 0.03	
			Si_3_N_4_–TiN composites with 20 wt.% TiN	884 ± 32.6			317			4.62 ± 0.11	
			Si_3_N_4_–TiN composites with 30 wt.% TiN	785 ± 51.2			330			4.71 ± 0.05	

## 4. Osteogenesis Performance of Si_3_N_4_

### 4.1. In Vitro Cellular Response and In Vivo Study of Si_3_N_4_

Guedes e Silva et al. [59] confirmed that Si_3_N_4_ is non-toxic via in vitro an experiment by evaluating the cytotoxicity of Si_3_N_4_ sample extracts based on ISO 10993-5 (the Biological Evaluation of Medical Devices—Part 5: Tests for In Vitro Cytotoxicity). In addition, Neumann et al. [65] tested the cytotoxicity of five different qualities of standard industrial Si_3_N_4_ by using a L929-cell in a direct contact culture assay. The results showed that there was no cytotoxicity of Si_3_N_4,_ and the cell morphology of Si_3_N_4_ group remained the same as that of the control group. Furthermore, Guedes e Silva et al. [66] investigated the biocompatibility of Si_3_N_4_ via an in vivo rabbit tibia model and confirmed no adverse reactions from the surrounding tissues after two months of implantation. Consistent results regarding the biocompatibility of Si_3_N_4_ have been reported [13], and to date, there is no controversy regarding the biocompatibility of Si_3_N_4_. Additionally, a number of studies have shown that Si_3_N_4_ has a favorable osteogenic effect [67].

**Figure 3:**
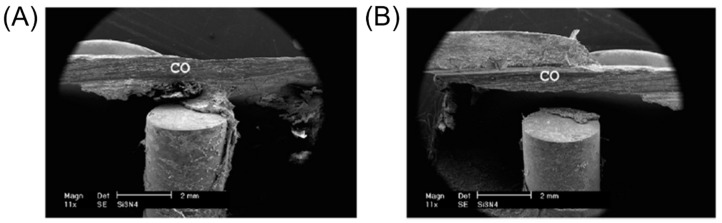
Scanning electron microscopy (SEM) images of the Si_3_N_4_ implants. (**A**) The bone bridge was formed extending to the implant surface for implant installed into the distal position of the rabbit’s tibia. CO = Compacta. (**B**) The bone bridge was not formed for the implant installed into the proximal position of the rabbit’s tibia. CO = Compacta. The image is reprinted with the permission from ref. [68]. Copyright 2007 Elsevier Publishing Group.

Cappi et al. [53] reported that Si_3_N_4_ fabricated by both hot-pressing and pressureless sintering was biocompatible with human mesenchymal stem cells (hMSC) and led to osteogenic differentiation of hMSC. In parallel to these in vitro experiments, various in vivo animal models were used to assess the bone-forming ability of Si_3_N_4_. Guedes e Silva et al. [68] implanted Si_3_N_4_ samples into the tibia of rabbits and analyzed the implant and the surrounding bone tissue after two months of implantation (Figure 3). The results illustrated that bone growth occurred mainly in the cortical region and varied in the distal and proximal regions of the implant. In the distal position where the distance between original compacta and implant was small, bone bridges were formed from the original bone tissue toward the implant surface, thus suggesting that Si_3_N_4_ is osteoconductive. Moreover, Howlett et al. [69] investigated the in vitro and in vivo effects of Si_3_N_4_ on rabbit skeletal cells and tissue. In their in vitro study, bone marrow stromal cells (MSC) attached and differentiated only near the Si_3_N_4_ samples but not within pores. However, their in vivo test results showed that Si_3_N_4_ implants were permeated by new mature bone after implantation in femoral bone marrow cavities for three months. This study demonstrated the potential of Si_3_N_4_ for applications in bone tissue engineering. In addition, another in vivo study in which Si_3_N_4_ was implanted into the tibia of rabbits showed that different types of tissue were identified on the implant’s surface, such as non-calcified matrix containing osteoblasts; a lamellar bone tissue containing osteocytes and osteons; and the presence of collagen III, which may transform to collagen I or remain as a fibrous tissue [66]. Furthermore, the authors found that bone tends to grow first in the cortical region.

Webster et al. [70] measured resistance for implant push-out in a rat model after 3 months post-implantation, and the quantitative results showed that Si_3_N_4_ had significantly higher bone attachment and growth compared to polyether ether ketone (PEEK) and a titanium alloy. Neumann et al. [71] also verified the biocompatibility of Si_3_N_4_ in animal models. In their study, thirty-six Si_3_N_4_ cylinders were implanted (i.e., “press fit”) into the lateral femur condyles of New Zealand male rabbits, and aluminum oxide biomaterials were chosen as the control group. After 2 months of implantation, there was no local or generalized immune-inflammatory reaction. Instead, histological analyses showed significantly higher bone-implant contact for Si_3_N_4_ than for aluminum oxide, although there was no statistical difference in digital imaging analysis. This investigation suggested that Si_3_N_4_ showed favorable biocompatibility in vivo and better osseointegration than aluminum oxide. In another experiment, they also implanted Si_3_N_4_ into frontal bone defects in mini-pigs for 3 months and confirmed new bone tissue formation in direct contact with the implants by CT and MRI scans [72]. The in vivo results from different animal models with Si_3_N_4_ ceramic are summarized in Table 2.

### 4.2. Mechanism and Influence Factors of Osteogenesis Properties

Recently, many researchers have focused on how Si_3_N_4_ accelerates bone repair and induces osseointegration. However, elucidating the clear mechanism behind its osteogenic behavior remains a daunting challenge.

Intuitively, there are two main elements in Si_3_N_4_, namely, Si and N. On the one hand, silicon itself is not biologically active. However, nanoscale etching can form grooved silicon surfaces and increase its biodegradability with the release of silicic acid. Its release can inhibit osteoclastic bone resorption by antagonizing the activation of signal transductors [75]. On the other hand, the composition of N is important for biogenic activation. Therefore, many researchers assumed that both Si and N may contribute to the osteogenic ability of Si_3_N_4_. Pezzotti et al. [76] investigated the effect of silicon and nitrogen ions released from the surface of Si_3_N_4_ through Raman, Fourier transform infrared spectrum, X-ray photoelectron spectroscopy, and histologic analyses. The results illustrated for the first time that Si and N elements can stimulate both osteosarcoma and mesenchymal cell differentiation and osteoblastic activity, thus increasing the rate of bone growth. Zanocco et al. [77] treated Si_3_N_4_ with a high-power pulsed laser to produce Si_3_N_4_ with different surface stoichiometries. Then, human osteosarcoma cells (SaOS-2) were used to test the osteogenic behavior of samples with different Si/N atomic ratios. The results demonstrated that increasing the nitrogen content promoted cell proliferation. This study also showed that both Si and N play synergistic roles in osteogenesis. 

When Si_3_N_4_ is placed in aqueous solution, the covalent cleavage of Si-N bond occurs. The spontaneous release of ammonia coupled with the formation of a hydrated layer of silicon dioxide on the surface (as shown in Equations (1) and (2)) will synergistically interact with the human body [78].

(1) $${\mathrm{Si}}_{3}N_{4}+6\;H_{2}O\to 3\;{\mathrm{SiO}}_{2}+4\;{\mathrm{NH}}_{3}$$

(2) $${\mathrm{SiO}}_{2}+2\;H_{2}O\to \mathrm{Si}{\left(\mathrm{OH}\right)}_{4}$$

On the one hand, ammonia-modified surfaces promote osteoblast activity by allowing covalent coupling of proteins [79]. On the other hand, the release of ammonia as shown in Equation (1) can also facilitate the formation of silanol groups, thus enhancing the overall formation of hydroxyapatite nuclei [80,81].

PO_4_^3−^ groups in Ca-apatite has been commonly recognized as a crucial reason for the affinity with NH^3+^ moieties of the peptide. Conversely, SiO_4_^4−^ groups have also been reported as a major factor for osteogenesis in some biomaterials, such as bioactive glass and calcium silicate. Similarly, for Si_3_N_4_, the formation of silanol groups (SiOH) can promote interface mineralization. Planar and the cyclic silicate trimer (Si_3_O_9_) includes three silanols aligned at 60° angles to each other, providing a stereochemical match for O atoms bound to Ca^2+^ on the (001) face of hydroxyapatite. Although silanol groups can provide negatively charged surfaces, the affinity of NH^3+^ with silanols was very low due to the acidic characteristics of both groups [82]. However, Si can stabilize bone matrix molecules and prevent enzymatic degradation by binding to glycosaminoglycan macromolecules and promoting the crosslink formations between proteoglycans and collagen [29].

**Figure 4:**
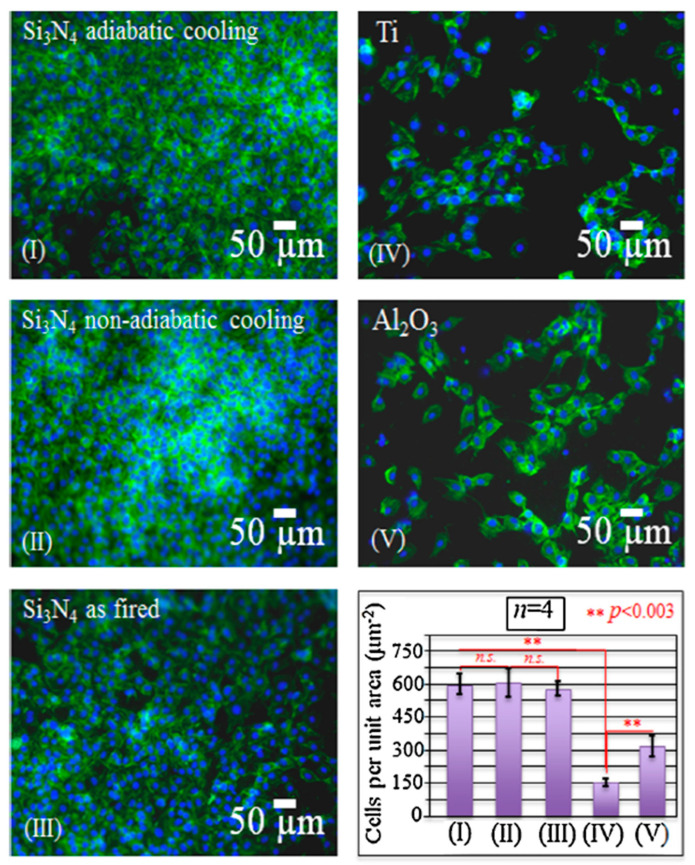
The fluorescence images of KUSA-A1 cell adhesion on different investigated samples (cf. labeling in inset) and the results of cell counting analyses on different samples (bottom right draft). The numbering in this latter plot corresponds to the numbers shown in each micrograph. The image is reprinted with the permission from ref. [29]. Copyright 2017 Elsevier Publishing Group. n.s.: Not significant.

**Figure 5:**
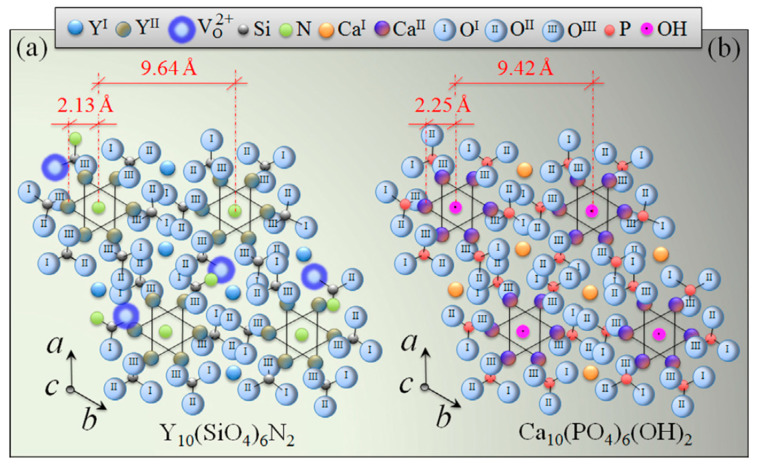
The (0001) view of the hexagonal apatite structures of N-(**a**) and Ca-(**b**) apatites. The image is reprinted with the permission from ref. [29]. Copyright 2017 Elsevier Publishing Group.

**Figure 6:**
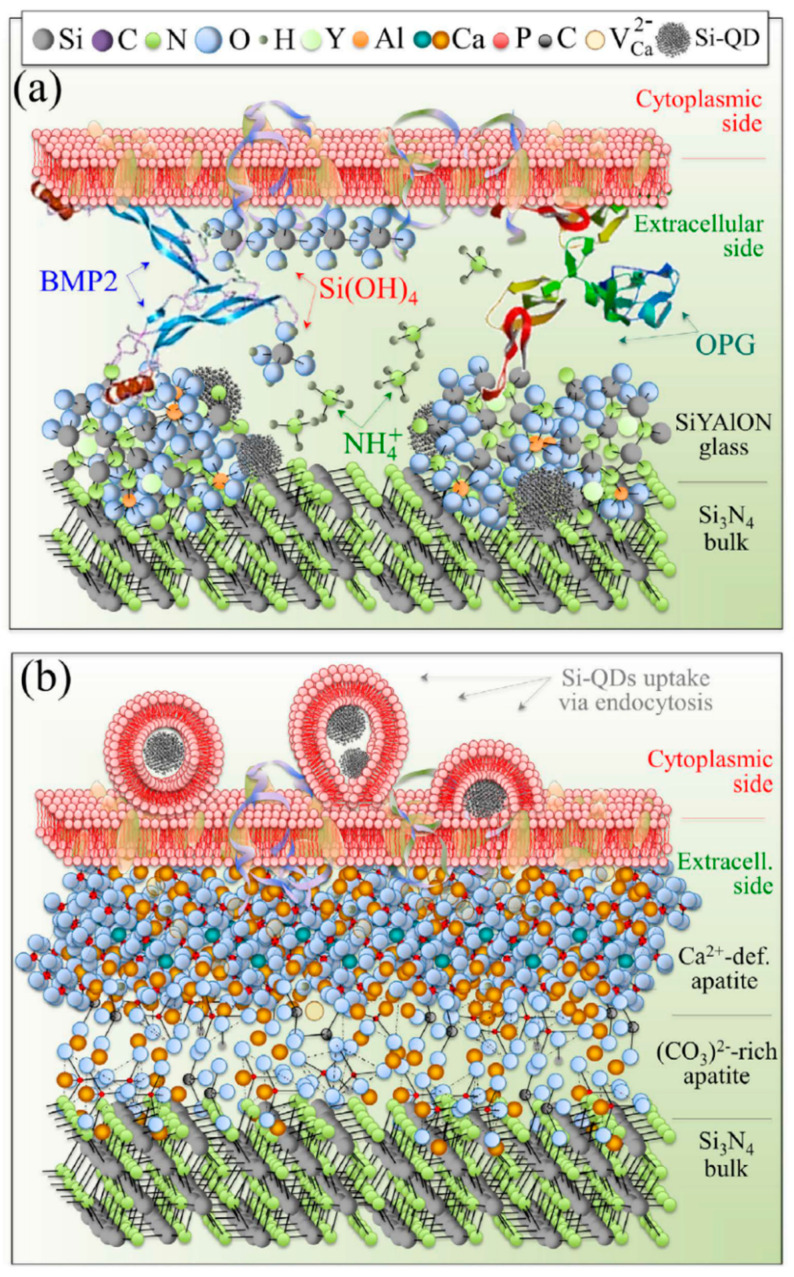
Schematic illustrations of the hypothesized metabolic activity of SaOS-2 cells on the surface of N2-annealed Si_3_N_4_: (**a**) the early-stage interaction with elementary molecules emitted from the Si_3_N_4_ surface and release of OPG and BMP2; and (**b**) the deposition of carbonate apatite and hydroxyapatite layers with concurrent endocytosis of Si-QDs. The image is reprinted from ref. [15].

Surface functionalization is one of the most effective techniques to improve material properties for specific applications. In particular, chemical surface modifications have a strong influence on cellular response and protein adsorption [83]. To some extent, progenitor cells can sense the intrinsic characteristics of the surface and receive signals released from the biomaterials. Usually there are electrically charged ionic sites and quantum dots distributed on the surface of bioceramics, which can influence cell metabolism or prompt protein folding on their surface. Specifically, for Si_3_N_4_, surface phases and off-stoichiometry can be effective factors in cell/surface interaction processes. The surface chemistry of Si_3_N_4_ can be influenced by different pressurized heat-treatments and by sintering additives. Yttrium oxide (Y_2_O_3_) is the most popular commercial additive, and there are also crystalline phases formed from the gain-boundary by products in Si_3_N_4_, such as N-apatite (Y_10_(SiO_4_)_6_N_2_), M-melilite (Y_2_Si_3_N_4_O_3_), N-YAM (Y_4_Si_2_O_7_N_2_), and N-wollastonite (YSiO_2_N) [29,84]. Pezzotti et al. [29] examined the interaction between various chemically modulated Si_3_N_4_ surfaces and murine mesenchymal progenitor cells (Figure 4 and Figure 5). It was found in their study that thermal-treatment coupled with adiabatic cooling could lead to partial coverage with N-apatite (Y_10_(SiO_4_)_6_N_2_), while non-adiabatic cooling resulted in full coverage by mostly yttrium silicate (−Y_2_Si_2_O_7_). These surface phases were found to induce the differentiation of murine mesenchymal progenitor cells into osteoblasts. Although the N-apatite coverage was limited, the final amount of bone formation was comparable to full coverage by mainly Y-disilicate phase. As-fired samples before a N_2_-annealing cycle were also prepared in this study. They compared the N-apatite phase Si_3_N_4_ samples with aluminum oxide and a titanium alloy. A quantification method to monitor KUSA-A1 mouse osteoblast cell proliferation was used to calculate the number of cells per unit. The results showed that the three Si_3_N_4_ groups had similar cell numbers but four-fold and two-fold higher than those of the titanium alloy and aluminum oxide. However, the Gla-/Glu-osteocalcin and growth factor (IGF-1) results indicated that the two groups with modified surface stoichiometries had higher cell differentiation and apatite growth. This same research group also examined the reciprocity between Si_3_N_4_ and living cells as a consequence of the off-stoichiometric chemical nature of its surface at the nanometer scale to see how Si_3_N_4_ affected cellular signal transduction functions and differentiation mechanisms (Figure 6) [15]. The study identified that some peculiar chemical agents (i.e., NH^4+^, H_4_SiO_4_, and Si-QDs) residing on the surface of Si_3_N_4_ ceramics affected the metabolic activity of living SaOS-2 cells.

The response of mesenchymal cells to a rough surface is partially determined by protein adsorption. A rough surface increases surface area, which leads to an increase in protein adsorption. In addition, the effect of roughness on protein adsorption is stronger for large proteins (e.g., fibrinogen) in comparison with small protein such as albumin, which is probably due to the enhanced unfolding of large proteins on rough surfaces. Additionally, increased related protein adsorption is correlated with enhanced osteoblast function. As the major protein component of natural bone, osteocalcin strongly links to the surface of hydroxyapatite along one face of its N-terminal helix via Gla-residues. A post-translational carboxylation of glutamic acid provokes these acid units into proteins and introduces an affinity for calcium ions, which is pivotal in the early stage of bone formation. Especially for bony apatite structure, peptides can bind to the Ca-apatite surface tightly due to its affinity with COO^−^ and NH_3_^+^ groups [29]. The surface of an engineered Si_3_N_4_ with Y_2_O_3_ sintering additives showed a folding preference of the peptide. Yttrium as a lanthanide cation shares some similar common properties with calcium ions pertaining to ionic radii, electrostatic interactions, and an affinity for oxygen ligands. Nevertheless, the crucial difference between Y^3+^ and Ca^2+^ is the anionic species they can bind due to their different charge. Remarkably, it has been reported that lanthanide ions showed higher peptide affinity compared to divalent metal ions [85]. As a result of these notions and investigations, Pezzotti et al. [29] postulated that Y^3+^ is a key factor leading to the rapid folding of osteocalcin onto the surface of Si_3_N_4_ with a Y_2_O_3_ sintering additive. 

The structure of ceramics greatly influences cell activities. Bone graft materials with a macroporous structure facilitate cell attachment, migration, the transportation of nutrients, and metabolic waste. Macropores (>100 μm) are especially necessary for bone ingrowth and vascularization [86]. Anderson et al. [74] developed a porous form of Si_3_N_4_ (namely, cancellous-structured ceramics) and quantified the extent and rate of bone ingrowth into this cancellous-structured Si_3_N_4_ implanted in the medial femoral condyle of sheep. The results showed that in some implants, the depth of newly formed bone was greater than 3 mm after 3 months in situ, implying that the porous structure is beneficial for achieving skeletal attachment. Additionally, combining Si_3_N_4_ with other bioactive materials (e.g., hydroxyapatite [87,88], bioglass [89,90]) to fabricate composite implants is a good strategy to achieve both desirable mechanical properties and bioactivity. For example, Amaral et al. [90] fabricated Si_3_N_4_/bioglass composites with a weight ratio of 70–30% and selected MG63 and human bone marrow cells to evaluate their in vitro osteogenic activity. The results showed that the composite accelerated the proliferation of MG63 cells and supported the differentiation of human bone marrow cells.

## 5. Antibacterial Properties of Si_3_N_4_

One of the most widely reported properties of Si_3_N_4_ is its antibacterial effectiveness. Once bacteria colonize an implant’s surface by forming a biofilm, these pathogens resist host immune mechanisms and systemic antibiotics. Consequently, orthopedic infections occur, resulting in implant loosening, device failures, and the non-healing of fractures. The continued growth of the biofilm can even lead to life-threatening conditions [91,92]. Moreover, bacterial infections of implants are highly complicated and multi-factorial. Infections are influenced by species of bacteria, types of implants, host immune variables, and so forth [93]. Strains such as Gram-positive *S. aureus* and *S. epidermidis*, and Gram-negative *P. aeruginosa*, *E. coli*, and *Enterococcus*, are frequently implicated in orthopedic implant infections. A common prevention method is to use perioperative antibiotic prophylaxis, but it is less effective in eliminating the risk of infections pertaining to implant surgery. The perioperative and latent infections rate remains relatively high, with reported rates of between 2.7% and 18%, and the cost of repeated surgery is high [94,95,96]. According to The Review on Antimicrobial Resistance (AMR), bacterial infections may cause up to 10 million deaths per year in 2050. Therefore, materials that can resist bacterial colonization and expression are intrinsically essential for orthopedic implants. 

Among clinically used implants (e.g., titanium alloys, PEEK, and stainless steel), Si_3_N_4_ has reported been highly effective in the majority of studies. Webster et al. [70] evaluated implanted Si_3_N_4_, PEEK, and titanium in an in vivo rat calvariae model and demonstrated the excellent anti-microbial properties of Si_3_N_4_. In their study, the percentage of histological bacterial counts on the implant surfaces was 0%, 21%, and 88% for Si_3_N_4_, titanium, and PEEK, respectively, three months after surgery. Similarly, Ishikawa et al. [97] examined the antimicrobial properties of stainless steel, titanium alloy, PEEK, and Si_3_N_4_. Si_3_N_4_ showed superior antibacterial performance among these materials. 

The adhesion of bacteria to materials is complex and is influenced by both the properties of materials (i.e., chemical composition, surface topography, and surface wettability) and species of bacteria. Specifically, surface roughness was mentioned as being exceptionally important for the antibacterial properties of Si_3_N_4_. Usually, machined materials (e.g., materials need to be ground flat) have a micron-rough surface. As-fired Si_3_N_4_ (e.g., no further processing after hot isostatic pressing) shows a nanotextured surface with randomly oriented acicular protruding grains. This results in a larger total surface area that may affect bacterial interaction. In the aforementioned study by Ishikawa et al. [97], they further compared the antibacterial property of as-fired and machined Si_3_N_4_ in vitro and in vivo. The former had fine nano- to micron-size anisotropic prismatic β-Si_3_N_4_ grains, while the machined Si_3_N_4_ had smooth surfaces. The results showed that the methicillin-resistant strain of *Staphylococcus aureus* (MRSA) could not adhere to native as-fired Si_3_N_4_ directly due to the macrophage clearance of the bacteria during biofilm formation, whilst non-uniformly machined areas demonstrated preferential bacterial adhesion to flatter surfaces. Additionally, the results of this investigation are consistent with those reported by Deborah et al. [91], in which PEEK showed the highest biofilm affinity and live bacteria counts, followed by titanium, polished Si_3_N_4_, and then as-fired Si_3_N_4_. Note that, especially for *Pseudomonas aeruginosa*, the live bacteria manifested on as-fired Si_3_N_4_ was approximately 1/60 of the bacteria found on PEEK. In addition, the protein adsorption studies demonstrated that as-fired Si_3_N_4_ had the highest adsorbed fibronection, vitronection, and laminin after 4 h among these test materials. Surface protein adsorption is relevant to the bacterial activity. According to this paper, increased vitronectin and fibronectin adsorption detrimentally affected the bacterial activity. Similar results and interpretations were also found in other studies [98,99,100]. However, this conclusion is controversial. Some have reported that fibrinogen or fibronectin facilitated the adhesion of bacteria through specific ligand-receptor interactions [101,102,103]. Truly, the relationship between protein adsorption and bacterial adhesion is difficult to understand due to various species of proteins and bacteria. Extensive studies should emphasize the interactive mechanisms of the bacteria, proteins, and implants. Nevertheless, it is generally accepted that bacterial adhesion can be decreased through engineering the surface topography of Si_3_N_4_.

**Figure 7:**
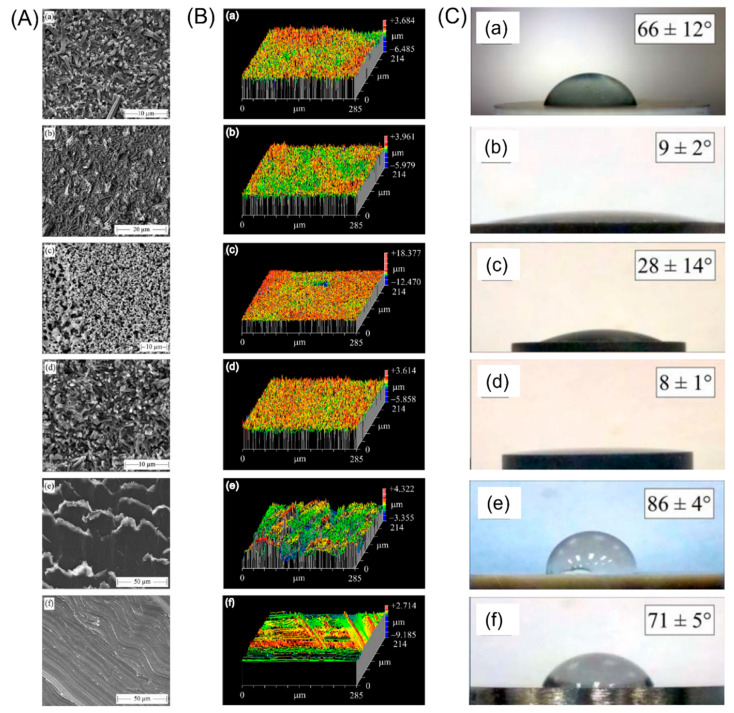
(**A**) SEM photographs; (**B**) white light interferometer oblique topographical plots; and (**C**) water droplet wetting angle photographs and measurements of (**a**) Af-Si_3_N_4_, (**b**) N2-Si_3_N_4_, (**c**) Gl-Si_3_N_4_, (**d**) Ox-Si_3_N_4_, (**e**) PEEK, and (**f**) Ti6Al4V. This image is adapted with permission from ref. [103]. Copyright 2017 Willey publishing group.

However, it should be noted that topographical factors alone do not significantly affect the behavior of bacteria. Other variables such as surface charge and surface energy also critically matter. Bock et al. [103] published a comparative study on biofilm formation for three biomaterials commonly used in spinal fusion surgery—Si_3_N_4_, PEEK, and a titanium alloy. In their study, they used different post-densification surface treatments to prepare four groups of Si_3_N_4_ with specific surface morphologies or chemistry, as shown in Figure 7: (1) as-fabricated Si_3_N_4_ without post-densification surface treatment; (2) N^2^-annealed Si_3_N_4_ consisting of samples with an increasing fraction of SiYAlON glass at the surface; (3) glazed Si_3_N_4,_ which was fully coated with SiYAlON; and (4) oxidized Si_3_N_4_ consisting of samples with increased an concentration of Si-OH groups on the surface. In conclusion, biofilm formation was found to be greatest on PEEK, followed by the titanium alloy and various Si_3_N_4_-treated samples. They discussed the mechanism of the bacteriostatic behavior of Si_3_N_4_ from different aspects. First, in relation to surface roughness, although a generally held axiom is that a rough surface contributes to bacteria adhesions, the nanostructured topography was found to have an opposite effect. In nature, it is well-known that the leaves of the lotus plant and the wings of the cicada have antibacterial properties. Their secret lies in the nano-rough pillar-like patterns on their surface [104,105]. Similarly, in this work, both machined titanium alloy and PEEK are more prone to biofilm formation compared to Si_3_N_4_ with its submicron- and nano-sized surface. Besides Si_3_N_4_, micron-sized arrays increased bacteria adhesion, whereas submicron patterns reduced adhesion for other materials in most cases [100,106,107]. Second, both surface charge and wettability are directly related to bacteria attachment. Since most bacterial genera have an overall negative surface charge, biomaterials with large negative zeta-potentials can inhibit bacteria adhesion through electrostatic repulsion [108]. Conversely, hydrophilic surfaces are not susceptible to bacteria attachment because the existence of adsorbed water on the surface is unfavorable for bacteria to gain a foothold [109]. Moreover, in this study, oxidized Si_3_N_4_ had the largest negative zeta-potential (≈−70 mV) at homeostatic pH and the lowest wetting angle (8±1°); therefore, it showed the overall lowest bacterial adhesion for both *S. epidermidis* and *E. coli* after two day’s incubation. Besides, the release of NH_3_ from the Si_3_N_4_ surface can increase the local pH (≈8.5), thus preventing bacteria adhesion and disrupting their cellular metabolism [102,110].

In addition, the low wear coefficient of Si_3_N_4_ also contributes to its anti-bacterial properties. Some studies have shown that the wear of abutments such as titanium implants generates particles and creates an ideal condition for a bacteria-mediated inflammatory burst, thus leading to peri-implantitis [111,112]. However, Si_3_N_4_ not only has a high wear resistance, but its wear particles are potentially soluble in biological fluids [113].

**Figure 8:**
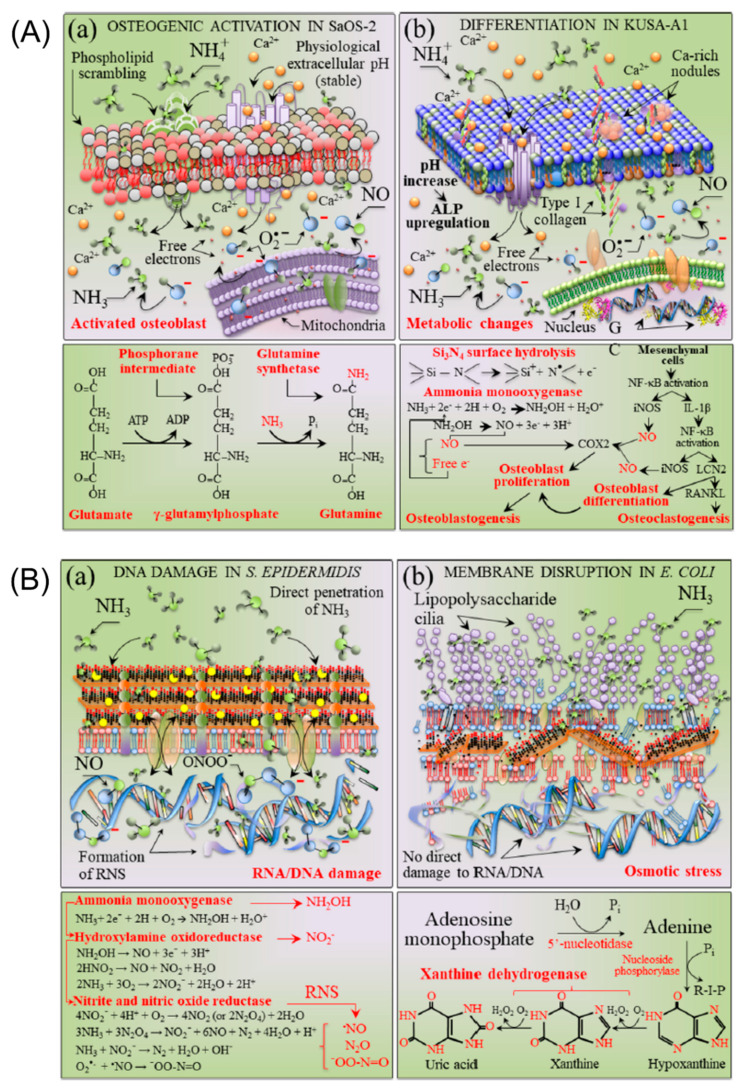
(**A**) Schematic diagrams of (**a**) SaOS-2 and (**b**) KUSA-A1 cell lines interacting with a Si_3_N_4_-activated aqueous environment. At the bottom of the respective draft, the cascade of the main chemical reactions involved with the substrate interaction is shown, which leads to glutamine synthetase and osteoblastogenesis in the (**a**,**b**) cases, respectively. In (**a**), the scrambled plasma membrane phospholipids PS, PC, and PE are drawn in red, brown, and beige colors, respectively, while the mitochondrial membrane is depicted in a uniform violet color. In (**b**), the nucleus membrane is represented by the green color. (**B**) Schematic diagrams of (**a**) *S. epidermidis* and (**b**) *E. coli* bacterial strains interacting with a Si_3_N_4_-activated aqueous environment. At the bottom of the respective draft, the cascade of the main chemical reactions involved with the substrate interaction is shown, which leads to direct RNA/DNA damage upon direct penetration of ammonia and membrane disruption by osmotic stress in the (**a**,**b**) cases, respectively. The lower panels to the drafts in (**a**,**b**) show the main chemical reaction, leading to RNS formation at the S. epidermidis/substrate biomolecular interface and the main path of metabolic stress detected in *E. coli* on the Si_3_N_4_ substrate, respectively. This image is reprinted with permission from ref [114]. Copyright 2019 ACS publishing group.

From the above discussion, silicon nitride was found to promote the healing of bone tissue but inhibit the proliferation of bacteria. These concurrent discoveries lead to a conundrum: how can Si_3_N_4_ be both beneficial to osteoblast cells and detrimental for bacteria and viruses? Pezzotti et al. [114] provided an answer after thoroughly investigating the particular surface chemistry of Si3N4 in aqueous environments using chemical analysis, biological time-lapse data from living osteoblasts and bacteria, high-resolution microscopy, and in situ Raman spectroscopy. On the one hand, the Si ions flushed from surface silanols by osteoblasts provide valuable building blocks for the synthesis of new bone tissue. On the other hand, nitrogen-radical interfacial chemistry provides two seemingly opposing effects on eukaryotic and prokaryotic cells in a single chemical action. The peculiar elution kinetics of N and Si species makes the surface environment of Si_3_N_4_ toxic to bacteria and nutritious to eukaryotic cells, as a function of pH. The mechanistic diagram and the chemical reactions for osteoblasts and bacteria due to their interaction with Si_3_N_4_ in an aqueous environment are shown in Figure 8. Figure 8A shows the cascade of the main chemical reactions of SaOS-2 and KUSA-A1 cell lines interacting with a Si_3_N_4_-activated aqueous environment, resulting in glutamine synthetase and osteoblastogenesis. However, in the bacterial strains S. epidermidis and E. coli interacting with an Si_3_N_4_-activated aqueous environment, the cascade of key chemical reactions leads to direct RNA/DNA damage upon direct ammonia penetration in S. epidermidis and membrane disruption by the osmotic stress in *E. coli*, respectively (Figure 8B). Based on this "smart" behavior of Si_3_N_4_, they referred to Si_3_N_4_ as a bioceramic with a gift.

## 6. Medical Application of Si_3_N_4_

**Figure 9:**
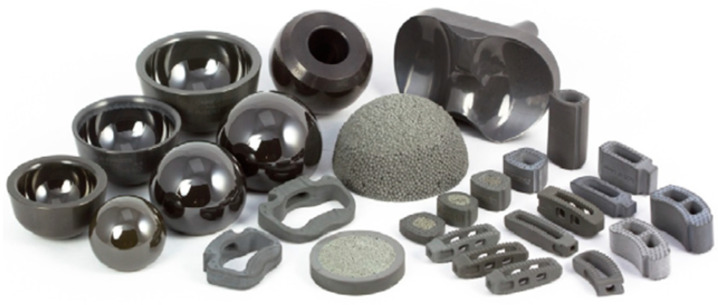
Representative spinal and reconstructive implants produced from biomedical Si_3_N_4_ (courtesy: SINTX Technologies Corporation). The image is reprinted from ref. [31].

Musculoskeletal disorders are the most popular health issues amongst the aging generation. Apart from that, the number of young people traumatized by traffic accidents and sports injuries is increasing. The knee, hip, and spine are the most frequently replaced body parts [2]. The demand of spinal fusion operations, total hip arthroplasty, and total knee arthroplasty is rising, and demand is expected to grow even faster in the future. Si_3_N_4_ has been explored as various prosthetic devices, from spinal fusion implants to joint replacements (Figure 9). Some orthopedic components made of Si_3_N_4_ have been commercialized. In addition, there are ongoing clinical studies using this biomaterial. To facilitate discussion, we have classified different applications of Si_3_N_4_ based on the types of surgeries.

### 6.1. Spinal Reconstruction Field

Symptomatic degenerative disorders of the intervertebral disc may cause chronic lower back pain, which limits patients’ activities and lowers their overall quality of life. Spinal fusion is a successful treatment for stabilizing degenerative segments to eliminate pain. Usually, patients are implanted with an intervertebral fusion cage, which bears direct axial loads, maintains the height of intervertebral and foraminal space, and eventually helps adjacent vertebrae fuse together. 

Currently, PEEK and titanium alloys are the two most popular materials used as fusion cages worldwide. However, complications, including migration or subsidence of PEEK cages, frequently occur. It was reported that the subsidence rate in patients with PEEK cages after lumbar interbody fusion was up to 14.3% [115]; however, titanium alloys often result in stress shielding within the body due to its high stiffness. The ideal intervertebral cage should fulfill the following requirements: (1) suitable size and geometry; (2) matched mechanical properties; and (3) high osseointegration ability. Due to its excellent mechanical properties and osteogenic behavior, Si_3_N_4_ cages or spacers are expected to allow for better fusion and lower complication rates than PEEK or titanium.

In addition, visualization on radiograms or CTs of the cage is critically important. This can help assess the exact position in the spine during and after the surgery and determine if fusion has occurred. Metal cages produce radiological artifacts on CT or MRI scans and PEEK is radiolucent, making radiographic evaluation more difficult, especially for the follow-up imaging. Si_3_N_4_, a partially radiopaque material, has propitious imaging properties and is free from artifacts on standard imaging techniques, such as MRI or CT. For instance, in Neumann’s study [72], implants made of Si_3_N_4_ showed no artifacts on CT and MRI scanning. Figure 10 from Ref. [116] shows the superior imaging properties of Si_3_N_4_ in comparison with PEEK, titanium, and trabecular metal. This implies that healthcare professionals are able to effectively assess the continuity of the surrounding tissue and bone in contact with the implant. 

**Figure 11:**
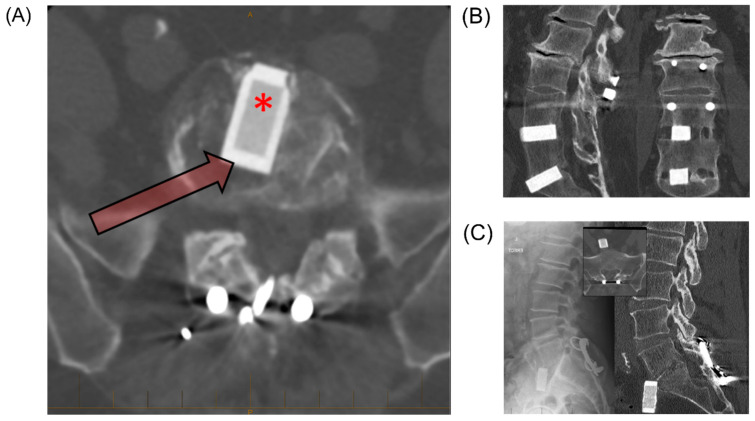
The 30-year follow up results of Si_3_N_4_ spacers. (**A**) The design characteristics of the Si_3_N_4_ implant. The computed tomography imaging shows the implant design, including a nonporous rim for load bearing (arrow), with a porous central core for early osseointegration (asterisk). (**B**) The sagittal computed tomography showing excellent implant position with some settling of the implant into the superior S1 end plate (left). The coronal computed tomography with fusion mass adjacent to the Si_3_N_4_ spacer, and osseointegration at the boneeceramic interface (right). (**C**) The anterior migration and dislocation of the Si_3_N_4_ implant with posterior revision. A 62-year-old man, currently asymptomatic and working full time as a publican. The standing radiograph with complete dislocation of the implant, and the subsequent posterior Hartshill rectangle fusion (left). The fusion of the sagittal computed tomography view through the L5/S1 segment, despite the implant having extruded anteriorly out of the disc space. (Insert) There is no reaction in the surrounding tissue, reiterating the fact that the Si_3_N_4_ reaction bonded implant material is biocompatible (right). This image is reprinted with permission from ref. [117]. Copyright 2018 Elsevier publishing group.

Pre-clinical laboratory bench tests and several clinical studies, including randomized and multicenter clinical trials, have been conducted using Si_3_N_4_ implants prior to and after applying for approval to the medical market. Si_3_N_4_ was first used as an anterior intervertebral spacer in a small clinical trial in Australia in 1986 under license by Sialon Ceramics Pty. Ltd. Follow-up results at time points of approximately 1, 5, 10, and 30 years have been presented [14,117]. In the 1-year review of 25 patients involved in pain assessment, more than half of the patients reported a substantial reduction in pain. The 5-year review of 22 patients showed very high overall satisfaction, with intervertebral bone fusion observed in almost all cases. Moreover, the occurrence of interspace collapse was much lower with the Si_3_N_4_ implants than the autologous bone grafts, although there was slippage in 1 patient, subsidence in 2 cases, loosening in 1 case, and the indication of reaction with the implant in 2 cases. The 10-year review of 16 patients showed that interbody bone fusion was maintained in all cases without subsidence, slippage, or reaction. However, it should be mentioned that progressive degeneration was observed in many cases, probably due to stress shielding. Five patients were followed for 30 years, using CT and standing radiography to obtain radiologic outcomes. Fusion and the osseointegration of surrounding tissue were achieved in 100% of the patients. However, 50% of patients had anterior slippages, and one patient had a complete extrusion of the implant (Figure 11). It is likely to be the result of a design that intentionally avoided additional fixation system (i.e., plates and screws). Overall, this long-term clinical study proved the biocompatibility, osseointegration, and bone formation ability of Si_3_N_4_.

New medical devices should be compared to the gold standard for their clinical performance. Kersten et al. [73] compared the bone formation ability of Si_3_N_4_ and PEEK in lumbar interbody fusion surgeries using a caprine model. The results demonstrated that Si_3_N_4_ spacers were not inferior to PEEK in bone-implant contact (BIC) and biodynamic stability, and they were superior in promoting arthrodesis. Apart from low back pain, radicular arm pain and paranesthesia (sometimes along with neck pain) are cervical radicular syndromes that result from disc herniation, with an annual incidence rate of circa 0.08% [116]. Mark et al. [116] presented the design of the CASCADE trial on the security and effectiveness of cancellous-bone-structured Si_3_N_4_ ceramic cages versus the gold standard PEEK cage for anterior cervical discectomy with fusion in 100 patients with cervical disc herniation and/or osteophytes. In this study, cancellous-bone-structured Si_3_N_4_ ceramic filled the center hole of the Si_3_N_4_ cage, providing a scaffold for bone ingrowth, which avoided harvesting iliac crest for autograft. The clinical and radiological results [118] of this single-blinded randomized controlled trial were reported in 2017. With 2 years follow-up, the results showed that there was no significant difference between Si_3_N_4_ and PEEK regarding recovery rates. 

Si_3_N_4_ received CE and FDA marketing clearance to be used as an interbody cage in 2008 based on animal studies and standard compliance [119]. Till now, few adverse events have been reported. In addition, numerous clinical results have reported that Si_3_N_4_ allows for higher fusion rates during the treatment of symptomatic degenerative lumbar disc disorders [120]. Nevertheless, slippage and dislodgement, which happen frequently for PEEK or CRFP cages [121,122], also occur in Si_3_N_4_ spinal implants, albeit at a low rate [14]. Theoretically, Si_3_N_4_ has the ability of osseointegration with the native vertebral bodies. If the bone adherence to the implant occurs at the same rate as the interbody fusion itself, then the implant is less likely to migrate. Conversely, factors such as bone morphogenetic proteins or bone marrow mesenchymal stem cells can also be placed in the Si_3_N_4_ implants during surgery to improve their osteoinductive properties.

Currently, there are a number of commercially available spinal spacers and spinal fusion cages, such as the Valeo® II PL/OL Interbody Fusion Device and the Valeo® C+CsC cervical interbody fusion cage. Many laws and regulations in the medical field were enacted to ensure the safety and effectiveness of the medical devices. Unlike laboratory studies, where researchers usually do experiments with a limited number of specimens to observe the response of specific materials, products fabricated at the company are mass-produced. Large-scale production can be more complex because raw materials and craft processes have a crucial influence on the quality of products. McEntire et al. [31] published a process validation and verification report on manufacturing intervertebral spinal spacers from a biomedical Si_3_N_4_. According to the results from Taguchi fractional factorial designed experiments, raw materials and firing conditions are the most critical process parameters for obtaining product properties, while compaction pressure and binder composition affect the product dimensions. The data showed that both individual component batches and multiple production powder lots fabricated by selected process parameters achieved tight statistical controls over dimensions with Cpk of >1.79 and a sigma level of ~5.4, and their average fracture toughness and flexural strength was 10.6 MPa/m^2^ and 995 MPa, respectively.

### 6.2. Joint Replacement Surgery

Arthritis, osteoporosis, and trauma are common health issues amongst the aging population, and treatment procedures can vary from medication, joint injections to surgical operation being the last resort [2]. Currently, the total replacement of the large natural joints, including the knee and hip, has widely evolved into surgical intervention for patients with intractable pain as the results of excessive joint degeneration [123]. Additionally, joint replacement surgery is expanding in the last decade. There are also discrepancies based on various markets. For instance, in Asian countries, smaller-sized bearings are used more commonly, whilst western countries prefer larger sizes. However, there is a common trend in both developing and developed countries—an increasing proportion of younger patients with joint replacements. Therefore, tougher and stronger joint prosthetics with a wide range of bearing configurations and sizes are being demanded to allow for surgical flexibility and withstand cyclic loading as much as possible [7]. 

It is of prime importance that the materials provide high wear resistance to minimize particulate debris associated with implant loosening, bone resorption, and even inflammation. In the case of Si_3_N_4_, the thin layer of orthosilicic acid (Si(OH)_4_) formed on the surface by scavenging oxygen from the tribolayer can act as a lubricant [78,124], which can be beneficial for long-term wear and implant longevity. The coefficient of friction for Si_3_N_4_ was found to be lower in humid air than in dry air or nitrogen in a study by Tomizawa et al. [125]. Later, they presented further convincing results that the coefficient of friction for self-mated Si_3_N_4_ in water decreased from circa 0.7 to a value less than 0.002 when the sliding speed went up to 60 mm/s [126]. Furthermore, the wear particles of Si_3_N_4_ can dissolve in PBS and bovine serum, and they thereby are expected to be resorbed in physiological systems with low immune response [113]. In conclusion, Si_3_N_4_ may be a potential candidate for joint replacement applications. 

**Figure 12:**
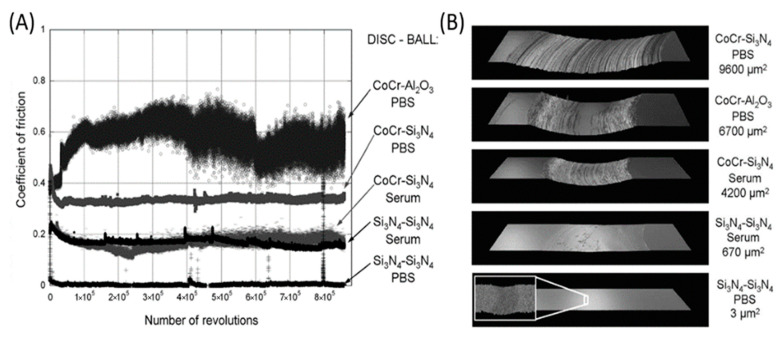
(**A**) The coefficient of friction as a function of number of revolutions in the pin-on disk test. (**B**) The optical profile images of worn discs and the calculated cross-section areas of the wear tracks. This image is reprinted from ref. [113].

**Figure 13:**
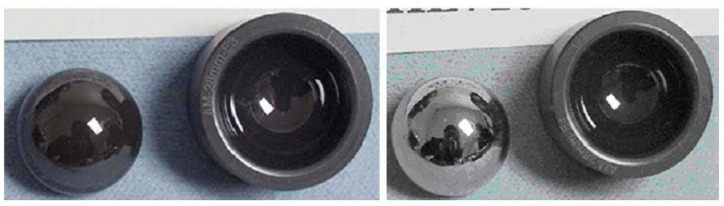
The Si_3_N_4_ bearings after 1 million wear cycles. The image is printed with the permission from ref. [64]. Copyright 2009 Elsevier Publishing Group.

The hip joint is the largest complex structure among hard tissue and soft tissue in the human body. It needs to undertake repetitive loads of large magnitude from daily activities. Unfortunately, the hip joint is particularly prone to failure due to sudden injury or degenerative diseases. Total hip arthroplasty (THA) is a common surgical intervention to treat such failure, using artificial materials to replace the bearing surface of both pelvis and femur [127]. The current material standard for joint replacement includes cobalt chromium (CoCr) metal alloys, ultrahigh molecular weight polyethylene (UHMWPE), highly cross-linked polyethylene (XPE). alumina (Al_2_O_3_), and zirconia-toughened alumina (ZTA) ceramics [128]. XPE has been especially recognized as the current “gold standard” in THA [123]. However, the tribo-corrosive process of CoCr alloys, the biological potential of polyethylene (PE) debris, and the component fracture and squeaking of ZTA ceramics cannot be ignored. An extensive effort has been made to improve the drawbacks of the commonly used materials, and the most logical option is to replace the bulk material with a new one that would minimize those problems. Based on the excellent mechanical properties and high wear resistance mentioned above, Si_3_N_4_ has gained increasing attention from many researchers. Johanna et al. [113] performed pin-on-disc wear tests with Si_3_N_4_ and CoCr discs sliding against Si_3_N_4_ or Al_2_O_3_ balls in PBS and bovine serum solutions. Figure 12 showed the coefficient of friction against the number of revolutions and optical profiles images of worn discs, as well as the calculated cross-section areas of the wear tracks. From this figure, we can clearly find that Si_3_N_4_-Si_3_N_4_ combinations showed relatively low coefficients of friction and areas of the wear track in both PBS and serum. Bal et al. [64] used Si_3_N_4_ to fabricate total hip arthroplasty bearings (Figure 13). The study demonstrated that Si_3_N_4_ cups produced lower wear rates when tested with either Si_3_N_4_ or CoCr femoral heads compared to Al_2_O_3_-Al_2_O_3_ bearings. Combining the reliability of CoCr femoral heads with the wear advantages of Si_3_N_4_ as a bearing couple has great potential for total hip arthroplasty.

**Figure 14:**
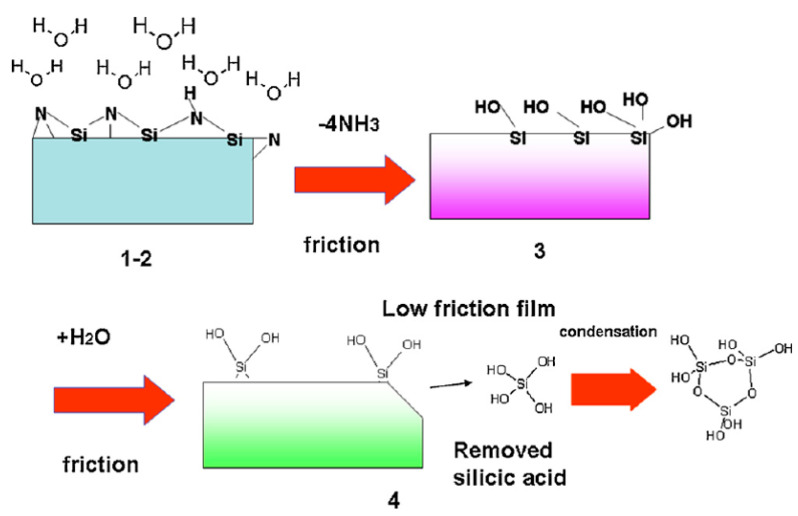
A sketch that shows an example of the tribochemical wear mechanism with water in four stages. The image is reprinted with permission from ref. [78]. Copyright 2012 Elsevier Publishing Group.

Although most THA surgeries are successful, the survival rates of hip prostheses after 10 years are unsatisfactory [129]. Recently, an article in the *Lancet* reported that a hip replacement can be expected to last 25 years in only 58% of patients who received THA [130]. Dr. Mallory, a pioneer in the field of total hip arthroplasty surgeries, illustrated this situation vividly, “All prosthesis will fail sometime. It is a race between the life of the patient and the life of the prosthesis”. Pezzotti et al. [127] systematically studied the ‘natural’ cycle of lifetime of hip implants when embedded in the physiological environment. He suggested that the lifetime of bioinert oxide ceramics (i.e., Al_2_O_3_ and ZrO_2_) was limited due to their oxygen activity in hydrothermal environment. However, as a non-oxide ceramic, Si_3_N_4_ has a relatively high wear rate initially under humid conditions, and then hydroxylated silicon oxide will be generated as the main product of tribochemical wear which can flatten the surface thus decreasing the friction level. Figure 14 schematically showed the tribochemical reactions. In order to decrease the initial wear rate, the pre-oxidization of the surface of Si_3_N_4_ was proposed. Rondinella et al. [131] investigated the wear behavior of ultrahigh molecular weight polyethylene (UHMWPE) pins against pristine or pre-oxidized Si_3_N_4_ femoral heads. The surface roughness was substantially lower for Si_3_N_4_ surfaces in pre-oxidized conditions owing to the formation of a thin silica/silanol layer. Such layers not only improved the tribological properties of Si_3_N_4_/UHMWPE couples but also delayed the oxidation of the UHMWPE counterpart. 

Arthritis primarily affects the surfaces of the joint and subchondral bone. Intuitively, it seems more logical to resurface the joint directly. The demand for minimal invasive implants such as hip resurfacing prostheses has dramatically increased in recent years, especially among younger patients who need skeletal implants. These innovative artificial joints can conserve as much of the native hard tissue of the patients as possible [132]. Si_3_N_4_, the non-oxide ceramic with high strength, has been recognized as a material candidate in the future, especially for highly loaded, thin-walled implants such as ceramic resurfacing hip prostheses [53]. Zhang et al. [133] investigated the mechanical reliability of Si_3_N_4_ as a new ceramic material in hip resurfacing prostheses. Through finite element analysis, they found that stress distributions in the femur bone with the implanted Si_3_N_4_ prosthesis were similar to those of healthy natural femur bone. To date, biological resurfacing of the hip joint is still in progress, but it could be an ideal choice for the treatment of hip osteoarthritis. Therefore, we look forward to more studies highlighting the possibility of Si_3_N_4_ as hip or knee resurfacing prosthesis.

It should be mentioned that although the potential use of Si_3_N_4_ in total joint arthroplasty has been studied and tested, they have not been approved by regulatory agencies. Additionally, there are still controversies of the wear performance of Si_3_N_4_ due to its surface oxide SiO_2_ layer, which may chip off, thus causing catastrophic 3rd-body wear (increased wear because of hard particles between softer articulating surfaces) [12]. In the research by Jahanmir et al. [134], they speculated that the oxide surface film needs to develop a wear scar of a specific size to achieve a high degree of conformity, thus reducing the contact pressure and maintaining the condition of low friction. Since the low friction behavior relies heavily on the presence of this lubricating film, breakdown, or damage of this film, could dramatically increase friction. Therefore, the exact impact of SiO_2_ flaking should be clarified before planning clinical follow-up studies. 

### 6.3. Other Clinical and Potential Use

In addition to spinal devices and total joint replacement, Si_3_N_4_ can be manufactured as plates or screws to treat fractures. Si_3_N_4_ prototype mini-fixation systems including mini-plates and screws were prepared and implanted in the frontal bone defects in 3 minipigs in the research of Neumann and co-workers [72]. These osteofixation systems showed satisfactory results in aspects of biocompatibility and mechanical stability and are potential options for osteosynthesis of the midface. However, it should be mentioned that simulated plate geometries regarding pullout forces at maximum load have limited safety in bending.

Dental implantology has revolutionized oral restoration procedures in recent years [135]. As for dental rehabilitation, titanium and zirconia as dental implants have shown high clinical success rates. However, new clinical challenges have emerged, especially peri-implantitis. This can damage the surrounding tissues and even lead to bone loss. Antibacterial implants may solve this problem. Si_3_N_4_ may be the ideal candidate due to its long-term stability and anti-bacterial properties. In the previously cited study on the bacteriostatic behavior of surface-modulated Si_3_N_4_, the author mentioned that dental bacteria research elucidated that the elution from Si_3_N_4_ generated peroxynitrite to penetrate the pathogen, thus downregulating bacterial metabolism and resisting bacterial expression [103]. Wasanapiarnpong et al. [136] fabricated white colored and high-density Si_3_N_4_ ceramics for dental core materials. The Si_3_N_4_ specimens were subsequently coated with two types of veneers: borosilicate glass and ZrO_2_ 5 wt.%-added borosilicate glass. Both Si_3_N_4_ and borosilicate glass have a low coefficient of thermal expansion, which is close to the natural human teeth. Additionally, the hardness values of both veneers are equivalent to those of human teeth ranging from 3 to 5 GPa. From the cytotoxicity test by the MTT assay, both the core and veneer ceramics are biocompatible with human gingival and periodontal ligament fibroblasts. Thus, this dental ceramic made of a Si_3_N_4_ core with borosilicate glass veneer may be a good choice for dental materials. However, more clinical data on Si_3_N_4_ as a dental implant are needed to discuss its efficacy in detail.

As we can see from this review, many groups have been actively conducting a lot of studies combining mechanics and biological aspects of Si_3_N_4_. However, it is still very challenging but rewarding to optimize the properties of Si_3_N_4_ and to integrate scientific technologies to apply Si_3_N_4_ materials to commercial products for clinical needs. Obviously, design methodology, near net shaping, machining, and quality control should also be carefully considered to produce highly reliable Si_3_N_4_ implants with possible lower manufacturing costs [54].

## 7. Conclusions

Considering its unique combination of properties, silicon nitride shows great potential for applications from both a basic research point of view and an industrial perspective. Si_3_N_4_ is a promising candidate for orthopedic implants due to its high strength, artifact-free imaging, and bio-responsiveness. It has several advantages over other commonly used biomaterials. For example, Si_3_N_4_ has a higher compressive strength than metallic biomaterials such as titanium and cobalt-chromium alloys, or biopolymers such as PEEK and UHMWPE, and a higher fracture toughness than some oxide bioceramics such as alumina. In addition to high fracture toughness, Si_3_N_4_ has a high wear resistance and a low coefficient of friction. Compared to metal implants, which produce radiological artifacts on CT or MRI scans, and PEEK, which is radiolucent, Si_3_N_4_ has favorable imaging properties and is free of artifacts on standard imaging techniques such as MRI or CT. In addition, compared to PEEK and titanium, the particular surface chemistry of Si_3_N_4_ in an aqueous environment leads it to promote bone tissue healing but inhibit bacterial proliferation. However, Si_3_N_4_ still has some disadvantages, such as brittleness, low energy dissipation, and high manufacturing cost. Currently, Si_3_N_4_ is already being used in arthrodesis devices in the cervical and thoracolumbar spine, and it is under consideration for approval in the joint arthroplasty and dental fields. Scientists and engineers have made great strides in expanding the use of Si_3_N_4_ for clinical applications and addressing various issues being faced by the industry today. Si_3_N_4_ has the potential to be microstructurally engineered and adapted to many applications, e.g., by grain size and morphology, grain boundary phase, or in a composite. We expect that further innovation of Si_3_N_4_ will come soon.

## Figures and Tables

**Figure 10:**
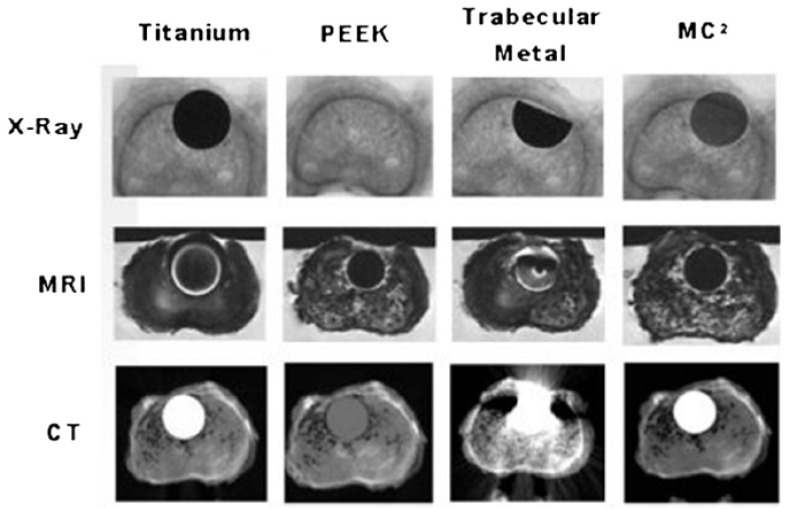
Si_3_N_4_, titanium, PEEK, and trabecular metal imaging characteristics in a human cadaveric vertebra. The image is printed with the permission from ref. [116].

**Table 2:** The in vivo results from different animal models withSi_3_N_4_ ceramic.

Author	Materials Used	Type of Implants	Animal Model	Control Group	Results	Ref.
Guedes e Silva et al.	Si_3_N_4_	/	Rabbits’ tibias	/	Bone growth occurred mainly in the cortical areas, and the bone bridge can be formed when the implants are installed into distal regions.	[68]
Kersten et al.	Si_3_N_4_	Lumbar interbody fusion implant	Caprine model	PEEK	Bone formation: the Si_3_N_4_ group (52.6%) was greater than PEEK (27.9%). BIC ratios and biodynamic stability: comparable.	[73]
Howlett et al.	Si_3_N_4_	/	Femoral marrow cavities in rabbit	/	In vivo test results showed that Si_3_N_4_ implants were permeated by new mature bone after being inserted into femoral massow cavities for three months.	[69]
Neumann et al.	Si_3_N_4_	/	lateral condyli of the femurs of New Zealand male rabbits	Aluminum oxide	Si_3_N_4_ shows good biocompatibility and presumably better osseointegration than Al_2_O_3_.	[71]
Neumann et al.	Si_3_N_4_	Miniplates and screws	Frontal bone defects in minipigs	/	These osteofixation system showed satisfactory results in terms of the aspects of biocompatibility and mechanical stability.	[72]
Anderson et al.	Cancellous-structured Si_3_N_4_	/	Femoral condyle of sheep	/	The results showed that in some implants, the depth of newly formed bone was greater than 3 mm after 3 months in situ, which meant that a porous structure is beneficial for achieving skeletal attachment.	[74]

## Data Availability

Not applicable.
